# Racial Dysmorphisms of the Ear Among the Tanzanian and Indian Populations: A Cross-Sectional Study

**DOI:** 10.7759/cureus.104232

**Published:** 2026-02-25

**Authors:** Juma Mwalimu, Justus A Kamara, Murtaza G Haiderbhai, Shabbir M Adamjee, Kalkidan K Mekoya, Dalila M Mwindadi, Mukasa Mohammed, Gibonce A Mwakisambwe, Ivony I Kamala, Pathan M Pathan

**Affiliations:** 1 Anatomy, St. Joseph University in Tanzania, Dar es Salaam, TZA; 2 Anatomy, Sri Ramachandra Institute of Higher Education and Research, Chennai, IND; 3 Public Health, Saifee Hospital Zanzibar Ltd, Zanzibar, TZA; 4 Medicine, Aenon Health Care, Dar es Salaam, TZA; 5 Family Medicine, Saifee Hospital Zanzibar Ltd, Zanzibar, TZA; 6 Surgery, Saifee Hospital Zanzibar Ltd, Zanzibar, TZA; 7 Internal Medicine, Saifee Hospital Zanzibar Ltd, Zanzibar, TZA; 8 Critical Care Medicine, Saifee Hospital Zanzibar Ltd, Zanzibar, TZA; 9 Obstetrics and Gynaecology, Saifee Hospital Zanzibar Ltd, Zanzibar, TZA

**Keywords:** ear biometric, ear helix reconstruction, external ear morphology, facial morphometry, racial dysmorphism

## Abstract

Introduction

The human ear exhibits significant morphological and morphometric variations across populations, influencing clinical practices, forensic identification, and psychosocial perceptions. This study aims to compare ear parameters between Tanzanian and Indian populations, assessing racial and sexual dysmorphisms.

Methodology

A cross-sectional study was conducted from March 2024 to February 2025 in Tanzania and India, involving 240 eligible participants aged 18-35 years (120 per country: 60 men and 60 women). Measurements, including ear length, width, lobule dimensions, concha parameters, and distances from the ear to the eye, mouth, and mandible, were taken using digital Vernier calipers and goniometers.

Results

Significant racial differences were observed in most ear parameters, with Indian ears generally larger than Tanzanian ears, except for right concha length, right concha width, and left concha width. Sexual dimorphism was more pronounced in the Indian population, with men showing larger parameters than women in most measurements, while Tanzanian men and women exhibited fewer significant differences. The ear's position relative to facial landmarks was significantly larger in the Tanzanian population. Lobule attachment types varied, with pendulous being the most common.

Conclusion

This study highlights significant differences in ear parameters between Tanzanian and Indian populations for both sexes. These variations are important, particularly in fields such as plastic and reconstructive surgery, prosthetic ear development, and otological treatments, where understanding population-specific anatomical differences is crucial for optimizing patient outcomes. In forensics, ear morphology is a reliable biometric marker for personal identification and aiding in criminal investigations and disaster victim identification.

## Introduction

The pinna is one of the most important defining features of the face; the shape, size, and location are also aesthetically significant [1]. Most of the dimensions of the auricle vary from birth to adulthood. The width of the ear matures between the ages of five and 11 years, and the length of the ear matures between the ages of 12 and 16 years [2]. Apart from hearing, the external ear (parts of the pinna) plays a role in surface landmarks and surface markings of the underlying structures, which are useful surgically, with an example of the lower border of the ear lobe being used as a landmark for the great auricular nerve during a nerve grafting procedure [3].

Over the decades, the human external ear has been among the tools used for personal identification; since 1890, when French police and biometric researcher Alphonse Bertillon applied anthropological techniques to law enforcement, he also introduced the mugshot method of photography for criminals by having their photographic record, allowing their identification [4]. The ear morphometrics and morphology can provide an additional tool for forensic studies/investigations [5].

Abnormal ear morphology, including ear prominence, and congenital anomalies are associated with psychosocial effects, including low self-esteem, anxiety, and depression before reconstruction [6,7]. Dimensions have also been found to differ according to age, ethnicity, side, and sex, making it useful for personal identification and as one of the facial parameters contributing to the appearance of the face [8]. Research on Indian populations has demonstrated that the morphometric parameters of the pinna, such as ear length (EL), width, and lobule dimensions, vary considerably among groups from North, Central, and South India. For instance, some studies indicate distinct patterns in ear shape and attachment types across these regions, with certain forms being more prevalent in specific geographical regions [1,9,10].

Even though literatures have documented morphometrics and morphology of the ear, there are no studies done in the Tanzanian population. Furthermore, comparison studies to describe the sexual dysmorphism of the ear among different races are limited. There are also no studies describing the position of the ear in relation to the angle of the eye, mouth, and mandible. This study aims to compare the morphometrics of ears between the Tanzanian and Indian populations, describing the sexual dysmorphism, as well as to determine the position of the ear from the lateral angle of the eye, angle of the mouth, and inferior angle of the mandible.

## Materials and methods

Study design and setting

A comparative cross-sectional analysis was executed over a 12-month period (March 2024-February 2025) to evaluate racial and sexual dimorphism in auricular morphology among Indian and Tanzanian cohorts.

Sample size estimation

A total of 240 participants were included in the study. Based on the mean difference of left EL above tragus (LELAT) between African (27.15) and Asian (30.21) populations from study article [11], and assuming a standard deviation of 5.1 from article [12], the sample size was calculated with a power of 90% and α error of 5%. The sample is calculated to be 60 in each group, that is, 60 women and 60 men from each country. That is 120 from each country.

Participant recruitment and selection

The study population comprised medical students aged 18 to 35 years. A simple random sampling method was employed to recruit a total of 240 participants, equally divided between the two countries (120 per country), with an equal distribution of 60 men and 60 women in each cohort. The study included volunteers with clinically normal ear morphology on both sides. Individuals with a history of ear trauma or disease, congenital ear malformations or craniofacial anomalies, extensively pierced ears, and a history of reconstructive ear surgery were excluded from the study.

Morphometric assessment and tools

Data were collected using standardized anthropometric tools using a digital Vernier caliper, which was calibrated to millimeters for high precision, and the values were later converted to centimeters for analysis. A goniometer was utilized for measuring angular parameters, and a standard scale was used for recording longer anatomical lengths. Participants were assessed in an upright sitting position, maintaining a neutral forward gaze. Measurements were taken for both the right and left ears. The measurements were made twice by different trained people, and the average of the results was used.

Morphometric parameters

**Figure 1:**
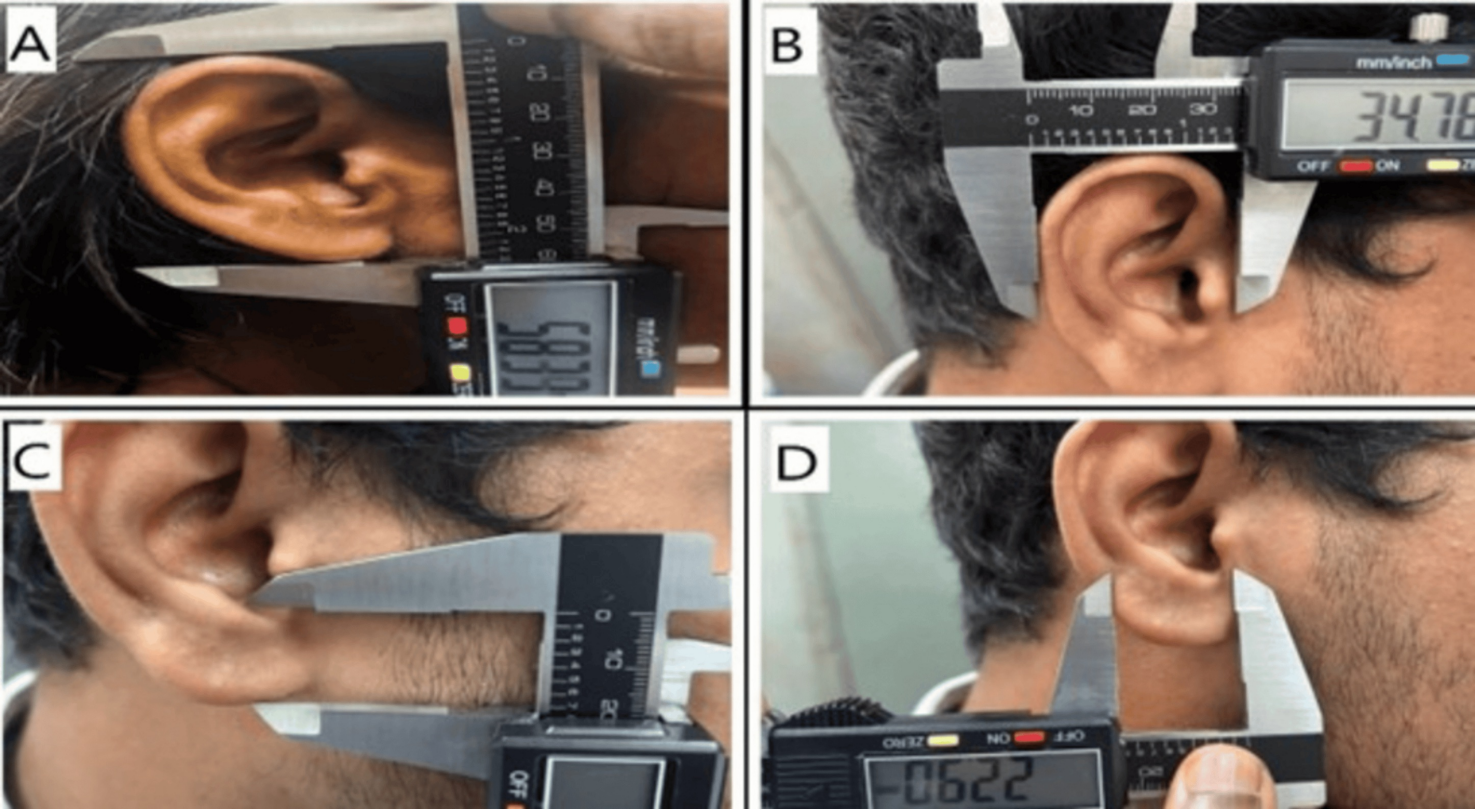
The images show how the measurements were done for the whole ear and the lobule Image (A) shows the measurement of the ear length (EL), image (B) shows the measurement of the ear width (EW), image (C) shows the measurement of the lobule length (LL), and image (D) shows the measurement of the lobule width.

**Figure 2:**
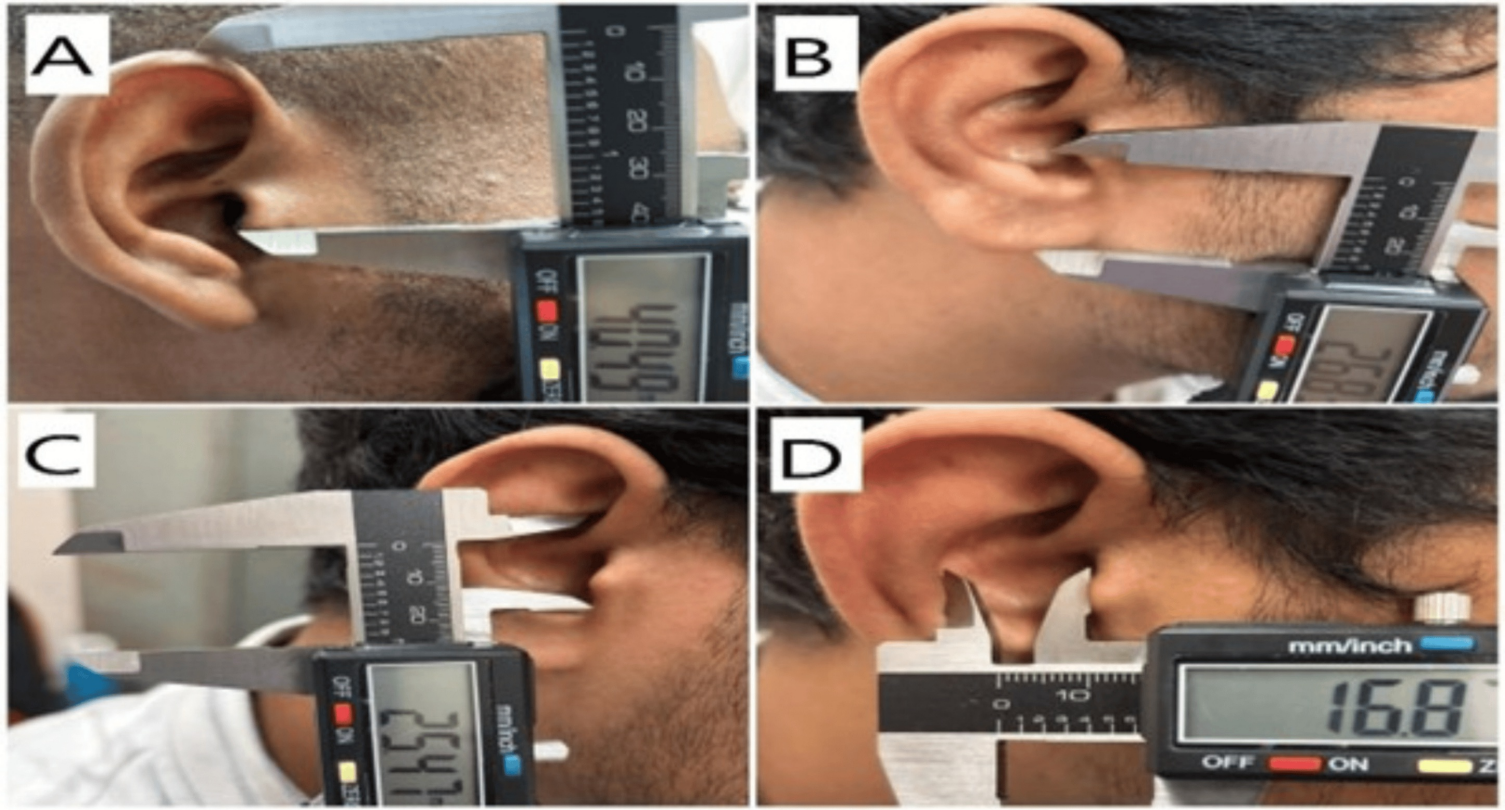
The images show the measurements were done for tragus and concha Image (A) shows the measurement of the ear length above tragus (ELAT), image (B) shows the measurement of the ear length below tragus (ELBT), image (C) shows the measurement of the concha length (CL), and image (D) shows the measurement of the concha width (CW).

The following parameters were measured and recorded for each participant: EL, ear width (EW), lobule length (LL), and lobule width (LW) (Figure 1) and EL above tragus (ELAT), EL below tragus (ELBT), concha length (CL), and concha width (CW) (Figure 2).

Other measurements included the lobule attachment angle (Ɵ) (categorized as tapered (acute, <90°), square (90°), or pendulous (obtuse, >90°)), distance from the root of the ear to the lateral angle of the eye (DATE), angle of the mouth (DMOTE), and angle of the mandible (DMATE). Detailed measurement protocols and the data collection tool used in this study are provided in the Appendices.

Ethical considerations

Institutional ethical clearance was secured prior to the commencement of the study. Informed consent was obtained individually from all participants before any measurements were performed.

Statistical analysis

Statistical analysis was conducted using the Statistical Package for the Social Sciences (SPSS). Descriptive statistics were used to summarize parameters. Racial and sexual differences were analyzed using t-tests. A p-value of <0.05 was considered statistically significant.

## Results

A total of 240 participants were included in the study, comprising 120 Tanzanians and 120 Indians, with equal representation of men and women in each population. All participants were aged between 18 and 35 years and had clinically normal external ears.

The comparison of overall ear morphometric parameters between Tanzanian and Indian participants has shown that significant racial differences were observed in most measured parameters. Indian participants demonstrated significantly larger mean values for EL, EW, LL, LW, ELAT, and ELBT on both sides when compared to Tanzanian participants (p < 0.05). However, no statistically significant differences were observed for right CL, right CW, and left CW between the two populations (Table 1).

**Table 1:** Comparison of ear parameters between the Tanzanians and Indians There are significant variations for both sides of the ear among the Tanzanians and Indians, with a p-value < 0.05, except for right and left width, left concha length, and width. Parameters are higher in Indians except for right concha length and width, and left concha width. All the measurements are in centimeters (cm). R: right; L: left; SD: standard deviation; N: number of samples

Parameters and side		Nationality	N	Mean	SD	Std. error mean	p-value
R	Ear length	Tanzanian	120	5.7277	0.44192	0.04034	<0.001
		Indian	120	6.2459	0.34376	0.03138	<0.001
	Ear width	Tanzanian	120	3.2043	0.31613	0.02886	0.054
		Indian	120	3.2755	0.24873	0.02271	0.054
	Lobule length	Tanzanian	120	1.5358	0.23534	0.02148	<0.001
		Indian	120	2.1695	0.23018	0.02101	<0.001
	Lobule width	Tanzanian	120	1.6619	0.19406	0.01772	<0.001
		Indian	120	1.9312	0.19166	0.01750	<0.001
	Concha length	Tanzanian	120	2.4297	0.35003	0.03195	0.034
		Indian	120	2.3510	0.20031	0.01829	0.034
	Concha width	Tanzanian	120	1.8160	0.27471	0.02508	<0.001
		Indian	120	1.6984	0.14688	0.01341	<0.001
L	Ear length	Tanzanian	120	5.5335	0.45069	0.04114	<0.001
		Indian	120	5.9263	0.25403	0.02319	<0.001
	Ear width	Tanzanian	120	3.1085	0.33763	0.03082	0.389
		Indian	120	3.1479	0.36982	0.03376	0.389
	Lobule length	Tanzanian	120	1.5326	0.22482	0.02052	<0.001
		Indian	120	2.0589	0.22224	0.02029	<0.001
	Lobule width	Tanzanian	120	1.6372	0.19100	0.01744	<0.001
		Indian	120	1.8692	0.18982	0.01733	<0.001
	Concha length	Tanzanian	120	2.3513	0.31028	0.02832	0.820
		Indian	120	2.3585	0.15073	0.01376	0.820
	Concha width	Tanzanian	120	1.7803	0.27013	0.02466	0.798
		Indian	120	1.7730	0.15345	0.01401	0.799

Sex-based comparisons within the Tanzanian population showed that Tanzanian men exhibited slightly higher mean values for several ear parameters compared to women; however, only a limited number of these differences reached statistical significance (p < 0.05), indicating minimal sexual dimorphism within this population (Table 2).

**Table 2:** Comparison of ear parameters between Tanzanian males and females There are no significant variations for both sides of the ear among Tanzanians with a p-value > 0.05, except for ear width and left ear length. Parameters are higher in males than in females, except for left lobule length and left concha width. All the measurements are in centimeters (cm). R: right; L: left; SD: standard deviation; N: number of samples

Parameters and side		Sex	N	Mean	SD	Std. error mean	p-value
R	Ear length	Males	60	5.8052	0.46880	0.06052	0.054
		Females	60	5.6502	0.40237	0.05195	0.054
	Ear width	Males	60	3.3295	0.36520	0.04715	<0.001
		Females	60	3.0792	0.19062	0.02461	<0.001
	Lobule length	Males	60	1.5400	0.23502	0.03034	0.844
		Females	60	1.5315	0.23757	0.03067	0.844
	Lobule width	Males	60	1.6885	0.19548	0.02524	0.134
		Females	60	1.6353	0.19054	0.02460	0.134
	Concha length	Males	60	2.4413	0.33812	0.04365	0.717
		Females	60	2.4180	0.36403	0.04700	0.717
	Concha width	Males	60	1.8308	0.27184	0.03509	0.556
		Females	60	1.8012	0.27904	0.03602	0.556
L	Ear length	Males	60	5.6408	0.47255	0.06101	0.009
		Females	60	5.4262	0.40367	0.05211	0.009
	Ear width	Males	60	3.2550	0.38712	0.04998	<0.001
		Females	60	2.9620	0.19082	0.02463	<0.001
	Lobule length	Males	60	1.5193	0.21621	0.02791	0.521
		Females	60	1.5458	0.23418	0.03023	0.521
	Lobule width	Males	60	1.6468	0.17844	0.02304	0.581
		Females	60	1.6275	0.20382	0.02631	0.581
	Concha length	Males	60	2.3787	0.26635	0.03439	0.337
		Females	60	2.3240	0.34888	0.04504	0.337
	Concha width	Males	60	1.7675	0.27484	0.03548	0.607
		Females	60	1.7930	0.26703	0.03447	0.607

In contrast, marked sexual dimorphism was observed within the Indian population. Indian men demonstrated significantly larger values for most ear parameters compared to Indian females on both the right and left sides (p < 0.05), suggesting a stronger influence of sex on auricular morphometry in this population (Table 3).

**Table 3:** Comparison of ear parameters between Indian males and females There are significant variations for both sides of the ear among Indians, with a p-value < 0.05, except for left lobule length. All the parameters are found to be higher in males than in females. All the measurements are in centimeters (cm). R: right; L: left; SD: standard deviation; N: number of samples

Parameters and side		Sex	N	Mean	SD	Std. error mean	p-value
R	Ear length	Males	60	6.4637	0.26465	0.03417	<0.001
		Females	60	6.0282	0.26807	0.03461	<0.001
	Ear width	Males	60	3.3970	0.21449	0.02769	<0.001
		Females	60	3.1540	0.22080	0.02851	<0.001
	Lobule length	Males	60	2.2427	0.20725	0.02676	<0.001
		Females	60	2.0963	0.23028	0.02973	<0.001
	Lobule width	Males	60	2.0443	0.12805	0.01653	<0.001
		Females	60	1.8182	0.17799	0.02298	<0.001
	Concha length	Males	60	2.4568	0.11195	0.01445	<0.001
		Females	60	2.2452	0.21357	0.02757	<0.001
	Concha width	Males	60	1.7377	0.15805	0.02040	0.003
		Females	60	1.6592	0.12409	0.01602	0.003
L	Ear length	Males	60	6.0420	0.16948	0.02188	<0.001
		Females	60	5.8107	0.27243	0.03517	<0.001
	Ear width	Males	60	3.2540	0.44666	0.05766	0.001
		Females	60	3.0418	0.23121	0.02985	0.002
	Lobule length	Males	60	2.0748	0.22726	0.02934	0.435
		Females	60	2.0430	0.21784	0.02812	0.435
	Lobule width	Males	60	1.9400	0.16867	0.02178	<0.001
		Females	60	1.7985	0.18450	0.02382	<0.001
	Concha length	Males	60	2.4077	0.10288	0.01328	<0.001
		Females	60	2.3093	0.17414	0.02248	<0.001
	Concha width	Males	60	1.8492	0.11867	0.01532	<0.001
		Females	60	1.6968	0.14701	0.01898	<0.001

Direct comparisons between Tanzanian and Indian men showed that Indian men had significantly greater mean values for most ear dimensions, including EL, EW, and lobule dimensions, compared to Tanzanian men (p < 0.05) (Table 4).

**Table 4:** Comparison of ear parameters between Tanzanian males and Indian males There are significant variations on both sides of the ear among Tanzanian and Indian males, with a p-value < 0.05, except for ear width and concha length. All the parameters are higher in Indian males except for right concha length and width, and left ear width. All the measurements are in centimeters (cm). R: right; L: left; SD: standard deviation; N: number of samples

Parameters and side		Nationality	N	Mean	SD	Std. error mean	p-value
R	Ear length	Tanzanians	60	5.8052	0.46880	0.06052	<0.001
		Indians	60	6.4637	0.26465	0.03417	<0.001
	Ear width	Tanzanians	60	3.3295	0.36520	0.04715	0.219
		Indians	60	3.3970	0.21449	0.02769	0.220
	Lobule length	Tanzanians	60	1.5400	0.23502	0.03034	<0.001
		Indians	60	2.2427	0.20725	0.02676	<0.001
	Lobule width	Tanzanians	60	1.6885	0.19548	0.02524	<0.001
		Indians	60	2.0443	0.12805	0.01653	<0.001
	Concha length	Tanzanians	60	2.4413	0.33812	0.04365	0.737
		Indians	60	2.4568	0.11195	0.01445	0.737
	Concha width	Tanzanians	60	1.8308	0.27184	0.03509	0.023
		Indians	60	1.7377	0.15805	0.02040	0.024
L	Ear length	Tanzanians	60	5.6408	0.47255	0.06101	<0.001
		Indians	60	6.0420	0.16948	0.02188	<0.001
	Ear width	Tanzanians	60	3.2550	0.38712	0.04998	0.990
		Indians	60	3.2540	0.44666	0.05766	0.990
	Lobule length	Tanzanians	60	1.5193	0.21621	0.02791	<0.001
		Indians	60	2.0748	0.22726	0.02934	<0.001
	Lobule width	Tanzanians	60	1.6468	0.17844	0.02304	<0.001
		Indians	60	1.9400	0.16867	0.02178	<0.001
	Concha length	Tanzanians	60	2.3787	0.26635	0.03439	0.433
		Indians	60	2.4077	0.10288	0.01328	0.434
	Concha width	Tanzanians	60	1.7675	0.27484	0.03548	0.037
		Indians	60	1.8492	0.11867	0.01532	0.038

Similar trends were observed when comparing Tanzanian and Indian women, with Indian women demonstrating larger ear measurements across most parameters (Table 5).

**Table 5:** Comparison of ear parameters between Tanzanian females and Indian females There are significant variations for both sides of the ear among Tanzanian and Indian females, with a p-value < 0.05, except for left lobule length. Parameters are higher in Indians except for concha length and width. All the measurements are in centimeters (cm). R: right; L: left; SD: standard deviation; N: number of samples

Parameters and side		Nationality	N	Mean	SD	Std. error mean	p-value
R	Ear length	Tanzanians	60	5.6502	0.40237	0.05195	<0.001
		Indians	60	6.0282	0.26807	0.03461	<0.001
	Ear width	Tanzanians	60	3.0792	0.19062	0.02461	<0.001
		Indians	60	3.1540	0.22080	0.02851	<0.001
	Lobule length	Tanzanians	60	1.5315	0.23757	0.03067	<0.001
		Indians	60	2.0963	0.23028	0.02973	<0.001
	Lobule width	Tanzanians	60	1.6353	0.19054	0.02460	<0.001
		Indians	60	1.8182	0.17799	0.02298	<0.001
	Concha length	Tanzanians	60	2.4180	0.36403	0.04700	<0.001
		Indians	60	2.2452	0.21357	0.02757	<0.001
	Concha width	Tanzanians	60	1.8012	0.27904	0.03602	0.003
		Indians	60	1.6592	0.12409	0.01602	0.003
L	Ear length	Tanzanians	60	5.4262	0.40367	0.05211	<0.001
		Indians	60	5.8107	0.27243	0.03517	<0.001
	Ear width	Tanzanians	60	2.9620	0.19082	0.02463	0.001
		Indians	60	3.0418	0.23121	0.02985	0.002
	Lobule length	Tanzanians	60	1.5458	0.23418	0.03023	0.435
		Indians	60	2.0430	0.21784	0.02812	0.435
	Lobule width	Tanzanians	60	1.6275	0.20382	0.02631	<0.001
		Indians	60	1.7985	0.18450	0.02382	<0.001
	Concha length	Tanzanians	60	2.3240	0.34888	0.04504	<0.001
		Indians	60	2.3093	0.17414	0.02248	<0.001
	Concha width	Tanzanians	60	1.7930	0.26703	0.03447	<0.001
		Indians	60	1.6968	0.14701	0.01898	<0.001

The position of the ear in relation to facial landmarks between the Tanzanian and Indian populations showed significantly greater distances from the lateral angle of the eye, angle of the mouth, and angle of the mandible to the root of the ear in Tanzanian participants when compared to Indian participants on both sides (p < 0.05) (Table 6).

**Table 6:** Comparison of the position of the ear in relation to the face among Tanzanians and Indians There are significant variations in the average position of the ear in relation to the face among Tanzanians and Indians, with a p-value < 0.05. All the parameters are found to be higher in Tanzanians than in Indians. All the measurements are in centimeters (cm). R: right; L: left; SD: standard deviation; N: number of samples

Parameters and side		Nationality	N	Mean	SD	Std. error mean	p-value
R	Distance from angle of eye to ear	Tanzanians	120	7.9592	0.44259	0.04040	<0.001
		Indians	120	7.6667	0.35677	0.03257	<0.001
	Distance from angle of mouth to ear	Tanzanians	120	11.3308	0.57858	0.05282	<0.001
		Indians	120	10.7517	0.80784	0.07375	<0.001
	Distance from angle of mandible to ear	Tanzanians	120	5.7001	0.52265	0.04771	<0.001
		Indians	120	5.4317	0.33406	0.03050	<0.001
L	Distance from angle of eye to ear	Tanzanians	120	7.9650	0.46808	0.04273	0.041
		Indians	120	7.8442	0.44435	0.04056	0.041
	Distance from angle of mouth to ear	Tanzanians	120	11.1108	0.59533	0.05435	<0.001
		Indians	120	10.6542	0.70662	0.06451	<0.001
	Distance from angle of mandible to ear	Tanzanians	120	5.7454	0.48201	0.04419	<0.001
		Indians	120	5.3933	0.41963	0.03831	<0.001

Tanzanian men exhibited slightly greater distances from facial landmarks compared to women, although only some parameters showed statistically significant differences (Table 7).

**Table 7:** Comparison of the position of the ear in relation to the face among Tanzanian males and females There are significant variations in the average position of the ear in relation to the face among Tanzanian males and females, with a p-value < 0.05, except for the distance from the angle of the mandible to the ear. All the parameters are found to be higher in males than in females. All the measurements are in centimeters (cm). R: right; L: left; SD: standard deviation; N: number of samples

Parameters and side		Sex	N	Mean	SD	Std. error mean	p-value
R	Distance from angle of eye to ear	Males	60	8.1133	0.48867	0.06309	<0.001
		Females	60	7.8050	0.32854	0.04241	<0.001
	Distance from angle of mouth to ear	Males	60	11.5183	0.55157	0.07121	<0.001
		Females	60	11.1433	0.54722	0.07065	<0.001
	Distance from angle of mandible to ear	Males	60	5.7417	0.48408	0.06249	0.386
		Females	60	5.6585	0.55955	0.07224	0.386
L	Distance from angle of eye to ear	Males	60	8.1183	0.50101	0.06468	<0.001
		Females	60	7.8117	0.37826	0.04883	<0.001
	Distance from angle of mouth to ear	Males	60	11.2483	0.57179	0.07382	0.011
		Females	60	10.9733	0.59114	0.07632	0.011
	Distance from angle of mandible to ear	Males	60	5.7800	0.42856	0.05533	0.433
		Females	60	5.7102	0.53230	0.06930	0.433

In the Indian population, men demonstrated significantly greater distances from facial landmarks compared to women across most parameters (p < 0.05) (Table 8).

**Table 8:** Comparison of the position of the ear in relation to the face among Indian males and females There are significant variations in the average position of the ear in relation to the face among Indian males and females, with a p-value < 0.05. All the parameters are found to be higher in males than in females. All the measurements are in centimeters (cm). R: right; L: left; SD: standard deviation; N: number of samples

Parameters and side		Sex	N	Mean	SD	Std. error mean	p-value
R	Distance from angle of eye to ear	Males	60	7.9150	0.23852	0.03079	<0.001
		Females	60	7.4183	0.27277	0.03521	<0.001
	Distance from angle of mouth to ear	Males	60	11.1583	0.83975	0.10841	<0.001
		Females	60	10.3450	0.52414	0.06767	<0.001
	Distance from angle of mandible to ear	Males	60	5.6050	0.27520	0.03553	<0.001
		Females	60	5.2583	0.29704	0.03835	<0.001
L	Distance from angle of eye to ear	Males	60	8.1183	0.31809	0.04107	<0.001
		Females	60	7.5700	0.37970	0.04902	<0.001
	Distance from angle of mouth to ear	Males	60	10.9917	0.62444	0.08062	<0.001
		Females	60	10.3167	0.62087	0.08015	<0.001
	Distance from angle of mandible to ear	Males	60	5.6333	0.39816	0.05140	<0.001
		Females	60	5.1533	0.28192	0.03640	<0.001

Comparisons between Tanzanian and Indian men revealed that Tanzanian men had significantly greater distances from facial landmarks to the ear compared to Indian men, except for the distance from the angle of the mandible to the ear and the left distance from the angle of the eye to the ear, where no significant differences were observed (Table 9).

**Table 9:** Comparison of the position of the ear in relation to the face among Tanzanian and Indian males There are significant variations in the average position of the ear in relation to the face among Tanzanian males and Indian males, with p-value < 0.05, except for the distance from the angle of the mandible to the ear and the left distance from the angle of the eye to the ear, which was found to be equal. All the measurements are in centimeters (cm). R: right; L: left; SD: standard deviation; N: number of samples

Parameters and side		Nationality	N	Mean	SD	Std. error mean	p-value
R	Distance from angle of eye to ear	Tanzanians	60	8.1133	0.48867	0.06309	0.006
		Indians	60	7.9150	0.23852	0.03079	0.006
	Distance from angle of mouth to ear	Tanzanians	60	11.5183	0.55157	0.07121	0.006
		Indians	60	11.1583	0.83975	0.10841	0.006
	Distance from angle of mandible to ear	Tanzanians	60	5.7417	0.48408	0.06249	0.060
		Indians	60	5.6050	0.27520	0.03553	0.060
L	Distance from angle of eye to ear	Tanzanians	60	8.1183	0.50101	0.06468	1.000
		Indians	60	8.1183	0.31809	0.04107	1.000
	Distance from angle of mouth to ear	Tanzanians	60	11.2483	0.57179	0.07382	0.021
		Indians	60	10.9917	0.62444	0.08062	0.021
	Distance from angle of mandible to ear	Tanzanians	60	5.7800	0.42856	0.05533	0.055
		Indians	60	5.6333	0.39816	0.05140	0.055

Similarly, Tanzanian women demonstrated significantly greater distances from facial landmarks compared to Indian women for all measured parameters (p < 0.05) (Table 10).

**Table 10:** Comparison of the position of the ear in relation to the face among Tanzanian and Indian females There are significant variations in the position of the ear in relation to the face among Tanzanian females and Indian females, with a p-value < 0.05. All the measurements are in centimeters (cm). R: right; L: left; SD: standard deviation; N: number of samples

Parameters and side		Nationality	N	Mean	SD	Std. error mean	p-value
R	Distance from angle of eye to ear	Tanzanians	60	7.8050	0.32854	0.04241	<0.001
		Indians	60	7.4183	0.27277	0.03521	<0.001
	Distance from angle of mouth to ear	Tanzanians	60	11.1433	0.54722	0.07065	<0.001
		Indians	60	10.3450	0.52414	0.06767	<0.001
	Distance from angle of mandible to ear	Tanzanians	60	5.6585	0.55955	0.07224	<0.001
		Indians	60	5.2583	0.29704	0.03835	<0.001
L	Distance from angle of eye to ear	Tanzanians	60	7.8117	0.37826	0.04883	0.001
		Indians	60	7.5700	0.37970	0.04902	0.001
	Distance from angle of mouth to ear	Tanzanians	60	10.9733	0.59114	0.07632	<0.001
		Indians	60	10.3167	0.62087	0.08015	<0.001
	Distance from angle of mandible to ear	Tanzanians	60	5.7102	0.53230	0.06930	<0.001
		Indians	60	5.1533	0.28192	0.03640	<0.001

## Discussion

This study reports significant racial differences in the morphology and morphometry of the pinna on both sides of the pinna between the Tanzanian and Indian populations. Most of the parameters measured were larger in Indian participants than in Tanzanian participants. Alexander et al. reported variations in ear parameters in three different races, with larger parameters for the Indian population, followed by Caucasians, and lastly Afro-Caribbeans [8]. Similar findings were reported between the Egyptian and Malaysian populations for both sexes [13].

As observed from our study, people from the same racial group may or may not vary significantly. Mumin et al. have reported a significant variation in the earlobe parameters among Indian Kenyans and Indigenous Kenyans; meanwhile, there were no differences among the Kikuyu and Luhyas people of Kenya [14]. However, in Nigeria, Indigenous people vary significantly among three ethnic groups [15]. Nedunuri and Patel reported significant differences between the Asian population and the African population, with measurements found to be higher in the Asian population than in the African population [11]. This is consistent with our observation, noting that Indians are part of the Asian population and Tanzanians are part of the African population.

Among the Tanzanian population, there are no variations in most parameters, and the parameters were higher in men than in women. In contrast, the Indian population exhibits significant variations in most parameters. In similar studies done in the Indian population, similar findings of gender differences have been observed with men having larger parameters than women across India [1,9-11]. Similar gender differences have been reported in the Sudanese, Nigerian, Thai, Turkish, and other populations [15-18].

The measurement of the distance from ear to eye, mouth, and angle of the mandible between the Tanzanian and Indian populations varies significantly, and the parameters are higher in Tanzanians than in Indians. Between the genders of the same population (among Tanzanians and Indians), these parameters also vary significantly, with more variation among Indians than among Tanzanians, and all the measurements are larger in men than in women. However, while comparing Tanzanian men and Indian men, 50% of the measurements showed no significant variations, while in women, all the parameters varied significantly. Similarly, it has been reported that facial morphometry differs significantly across ethnic groups, genders, and different ages [19,20]. It was noted that the major contributors were the jaw, cheek, brow, and nose [21].

As observed from this study, among the male gender population in Haryana and Himachal Pradesh of India, the facial parameters varied significantly for facial height, while there were no significant differences for facial width and facial index [22]. It has been reported that European and South American countries show more facial dysmorphism than African countries. These differences have been attributed to both allometric and non-allometric components, and the dysmorphism is not due to cross-cultural differences, masculinity, or sex differences in body height [23].

We have observed significant variations between the two study populations, as well as within the same population and between the two sides of the body. This shows that morphometry differs even among people of the same ethnic group [15]. Genetic factors are among the reasons for these differences. The genetic makeup among individuals differs; up to 93%-95% of the genetic composition in a given population is different, while the differences among the major groups range from 3% to 5% [24].

Even identical twins, despite sharing nearly identical DNA, are not perfectly alike. Subtle genetic variations and epigenetic modifications occur after conception, influenced by random cellular events despite exposure to a similar internal or external environment. These minute differences impact gene expression and development, leading to unique characteristics. This explains why even their facial features show minor left-right asymmetries. Thus, perfect replication in humans is biologically improbable [25].

The interaction of the ectodermal frontonasal process zone, bone morphogenic protein signaling, sonic hedgehog, and fgf8 proteins are factors for facial skeleton development during the prenatal period, while after-birth factors include structural, functional, genetic, and growth pattern interactions. It was identified that the Crf4 gene is responsible for smaller facial morphometry [26].

The variations in the single-nucleotide polymorphism among different populations existing in the genomic loci responsible for ear morphology and morphometry cause these racial differences in ear morphometry. Genomic loci 2q 12.3, 2q 31.1, 3q23, and 6q 24.2 are responsible for earlobe size, while loci 2q 12.3 and 2q31.1 are associated with earlobe attachment [14].

Congenital anomalies of the ear have been associated with social anxiety and loneliness [6]. Therefore, reconstructive/plastic surgeries involving the ear are expected to bring back joy. Since we have observed significant variations among populations, these studies provide standard reference values among different groups of individuals, which can be applied while doing reconstructive surgeries based on individuals' race [1,9,27]. This can also be applied in forensics as a biometric tool for individual identification, in criminal cases through mugshots [4], and in making ear tools like hearing aids and earphones, which can be customized in a race-specific manner.

Limitations and future research scope

Based on the results of this study and addressing the limitations, it is better that future research should focus on expanding the scope and depth of external ear morphometric studies across diverse populations and methodologies. One promising direction is to incorporate larger and more ethnically diverse samples within Tanzania and India, capturing the rich genetic and cultural heterogeneity of these regions. Beyond descriptive measurements, integrating genetic analyses could help identify genetic markers associated with specific ear morphologies, shedding light on the hereditary basis of ear shape.

## Conclusions

This study demonstrates significant racial differences in external ear morphometry between Tanzanian and Indian populations, as well as varying degrees of sexual dimorphism within each group. Indian participants exhibited generally larger auricular dimensions, while Tanzanian participants showed a more posterior positioning of the ear relative to facial landmarks. Sexual dimorphism was more pronounced in the Indian population than in the Tanzanian population.

These findings have important clinical and forensic implications. Population-specific auricular data are essential for optimizing outcomes in reconstructive and aesthetic ear surgery, prosthetic ear design, and otological procedures. Additionally, the documented morphometric variations support the use of ear morphology as a reliable biometric marker in forensic identification and anthropological research.

## Appendices

Data collection tool

Name:

Age:

Sex:

Nationality:

**Table 11:** Measurements done

S.No	Measurement	Right ear (cm)	Left ear (cm)
1	Ear length (EL)		
2	Ear width (EW)		
3	Lobule length (LL)		
4	Lobule width (LW)		
6	Lobule attachment angle (Ɵ)		
5	Ear length above tragus (ELAT)		
6	Ear length below tragus (ELBT)		
7	Concha length (CL)		
8	Concha width (CW)		
9	Distance from root of ear to angle of eye (DATE)		
10	Distance from root of ear to angle of mouth (DMOTE)		
11	Distance from root of ear to angle of mandible (DMATE)		
